# Short-term exposure to dimethyl fumarate (DMF) inhibits LPS-induced IκBζ expression in macrophages

**DOI:** 10.3389/fphar.2023.1114897

**Published:** 2023-02-01

**Authors:** Yong Zhang, Jingshu Tang, Yujun Zhou, Qiong Xiao, Qiuyu Chen, Hongyue Wang, Jiaqi Lan, Lei Wu, Ying Peng

**Affiliations:** State Key Laboratory of Bioactive Substances and Functions of Natural Medicines, Institute of Materia Medica, Chinese Academy of Medical Sciences and Peking Union Medical College, Beijing, China

**Keywords:** dimethyl fumarate, anti-inflammation, IκBζ, Nrf2, macrophage

## Abstract

**Background:** The pharmacological activity of dimethyl fumarate (DMF) in treating psoriasis and multiple sclerosis (MS) is not fully understood. DMF is hydrolysed to monomethyl fumarate (MMF) *in vivo*, which is believed to account for the therapeutic effects of DMF. However, previous studies have provided evidence that DMF also enters the circulation. Given that DMF is short-lived in the blood, whether DMF has a therapeutic impact is still unclear.

**Methods:** Lipopolysaccharide (LPS)-mediated RAW264.7 cell activation was used as a model of inflammation to explore the anti-inflammatory effects of short-term DMF exposure *in vitro*. Whole blood LPS stimulation assay was applied to compare the anti-inflammatory effects of DMF and MMF *in vivo*. Griess assay was performed to examined nitrite release. The expression of pro-inflammatory cytokines and transcription factors were measured by quantitative PCR (qPCR), ELISA and Western blot. Depletion of intracellular glutathione (GSH) was evaluated by Ellman’s assay. Luciferase reporter assays were performed to evaluate DMF effects on Nrf2-ARE pathway activation, promoter activity of *Nfkbiz* and mRNA stability of *Nfkbiz*. Binding of STAT3 to the IκBζ promoter were examined using Chromatin immunoprecipitation (ChIP) assay.

**Results:** Short-term exposure to DMF significantly inhibited the inflammatory response of RAW264.7 cells and suppressed LPS-induced IκBζ expression. Importantly, oral DMF but not oral MMF administration significantly inhibited IκBζ transcription in murine peripheral blood cells. We demonstrated that the expression of IκBζ is affected by the availability of intracellular GSH and regulated by the transcription factor Nrf2 and STAT3. DMF with strong electrophilicity can rapidly deplete intracellular GSH, activate the Nrf2-ARE pathway, and inhibit the binding of STAT3 to the IκBζ promoter, thereby suppressing IκBζ expression in macrophages.

**Conclusion:** These results demonstrate the rapid anti-inflammatory effects of DMF in macrophages, providing evidence to support the direct anti-inflammatory activity of DMF.

## 1 Introduction

Fumaric acid esters (FAEs) have been empirically applied to the treatment of psoriasis since the late 1,950 s (Wollina, 2011; Bruck et al., 2018). In 1994, an oral mixture of dimethyl fumarate (DMF) and three other FAE salts (Fumaderm^®^) was approved for psoriasis in Germany based on positive results from clinical trials (Nieboer et al., 1989; Nieboer et al., 1990; Nugteren-Huying et al., 1990; Altmeyer et al., 1994). Among the many FAEs in Fumaderm^®^, DMF has been demonstrated to be the main active ingredient (Nieboer et al., 1990; Mrowietz et al., 2017). In 2017, DMF monotherapy (Skilarence^®^) was approved by the European Medicines Agency as an oral therapy for moderate-to-severe chronic plaque psoriasis (Mrowietz et al., 2018a). Given the immunopathological similarity between psoriasis and multiple sclerosis (MS), DMF was also introduced for the treatment of relapsing-remitting MS (Fox et al., 2012; Gold et al., 2012; Kappos et al., 2014; Miller et al., 2015). In 2013, DMF was approved by the U.S. Food and Drug Administration (FDA) as a disease-modifying therapy (Tecfidera^®^) for relapsing-remitting MS (Venci and Gandhi, 2013). In recent years, DMF has become a first-line medication for MS.

Although DMF has achieved major success in the market, its mode of action is not yet fully understood. The prevailing view is that DMF serves merely as a prodrug (Mrowietz et al., 2018b). After oral intake, DMF is hydrolysed by gastrointestinal or plasma esterases to monomethyl fumarate (MMF), a major metabolite that modulates the immune system by activating both HCAR2 and Nrf2 (Mrowietz et al., 2018b). However, it was also reported that glutathione (GSH) conjugates of DMF (GS-DMS) and related metabolites were detected in human urine after oral intake of Fumaderm^®^ (Rostami-Yazdi et al., 2009). Subsequent studies in rats confirmed the presence of GS-DMS in plasma or even within the brain after gastrointestinal administration of DMF (Dibbert et al., 2013; Peng et al., 2016). Moreover, DMF-modified GAPDH proteins were detected in both the spleen and brain in DMF-treated mice (Kornberg et al., 2018), suggesting that DMF can modify not only GSH but also intracellular proteins with reactive cysteine residues *in vivo*. All these observations led to an additional hypothesis that some DMF molecules survive hydrolysis in the gut and rapidly enter the circulation to react with target proteins or GSH. Considering that DMF remains undetectable in the plasma after absorption (Litjens et al., 2004a; Rostami-Yazdi et al., 2010; Dibbert et al., 2013; Peng et al., 2016), exposure to DMF in the blood should be transient. However, whether transient exposure to DMF plays a functional role *in vivo* remains unknown. To the best of our knowledge, few studies have focused on the effects of short-term exposure to DMF. We believe that a study on this topic would be necessary to better understand the *in vivo* pharmacological activity of DMF and guide the future application of this molecule.

In this study, we examined the anti-inflammatory effects of DMF in short-term exposure to macrophages, a key disease mediator in the progression of experimental autoimmune encephalomyelitis (EAE) in mice and MS in humans (Mishra and Yong, 2016; Nally et al., 2019). We found that short-term exposure to DMF significantly inhibited the LPS-mediated inflammatory response of macrophages. Further mechanistic studies revealed that short-term exposure to DMF inhibited the expression of the NF-kappa-B inhibitor zeta (IκBζ) through multiple mechanisms, including the depletion of GSH, activation of Nrf2 and inhibition of constitutive STAT3 binding to the IκBζ promoter. The above results strongly suggest that DMF is likely to have a direct impact on immune cells *in vivo* before being hydrolysed to MMF, providing key evidence for the direct therapeutic effect of DMF.

## 2 Materials and methods

### 2.1 Reagents

DMF (222180250, Across), MMF (AK114632, ARK Pharm), and reduced glutathione (B96824, Innochem) were obtained through the Innochem reagent procurement platform. Lipopolysaccharide (LPS) from *Escherichia coli* 055:B5 (L2880, Sigma) and glutathione monoethyl ester (G1404, Sigma) were purchased from Sigma‒Aldrich. The non-covalent Nrf2 activator KI696 was kindly supplied by Dr. D.L. Yin of Institute of Materia Medica. The antibodies used for western blotting are listed in Supplementary Table S1 (Supplementary Material). The plasmid pGL4.13-Nfkbiz-promoter and pGL4.13-Nfkbiz-3UTR were constructed by YouBio (Changsha, China). The insertion sequences in both plasmids are listed in Supplementary Table S2 (Supplementary Material). The plasmid pSV40-Nfkbiz-3TUR, which harbours the mouse Nfkbiz ORF and its 3′-UTR elements, was constructed in our laboratory. The plasmid pcDNA3.1-mNrf2-6 × His was purchased from Miaolingbio (Wuhan, China). The plasmid pRL-TK was kindly provided by the group of Dr. Z.W. Hu of Institute of Materia Medica. All plasmids were transfected into cells using Lipofectamine 3,000 reagent (L3000008, Thermo Fisher Scientific).

### 2.2 Cell culture

The RAW264.7 cells were kind gifts from Dr. T.T. Zhang of the Institute of Materia Medica. Cells were cultured in complete DMEM containing 10% FBS (SE100-B, Vistech) and penicillin‒streptomycin (P1400, Solarbio). HEK293-ARE reporter cells harbouring pGL4.37 constructs were cultured in complete DMEM supplemented with 300 μg/mL hygromycin B (B29290, Innochem). The cells were subcultured every 2–3 days.

### 2.3 Generation of murine bone marrow-derived macrophages (BMDMs)

BMDCs were prepared from 6 to 8-week-old wild-type or Nrf2^−/−^ C56BL/6 mice according to the CSH protocol with modifications (Weischenfeldt and Porse, 2008). Briefly, bone marrow cells were collected from the femurs and tibias of mice and then cultured in L929-conditioned medium (L-medium) at an initial density of 2 × 10^6^ cells/mL. An equal volume of fresh L-medium was added on day 3. On day 6, half of the culture medium was replaced with fresh L-medium. Mature BMDMs were harvested at day 8 and treated as designed.

### 2.4 Cell viability assay

Cells were treated as designed in 96-well plates. Then, 10 μL of MTT solution was added to each well and incubated at 37°C for 4 h. The culture medium was carefully discarded, followed by the addition of 150 μL of DMSO per well to dissolve the purple formazan crystals on a shaker. The absorbance was measured at a wavelength of 570 nm.

### 2.5 Griess assay

The culture medium was added to an equal volume of 1% (w/w) sulfanilamide solution (S108473-100 g, Aladdin) and incubated at room temperature for 5 min. Another equal volume of 0.1% (w/w) N-(1-naphthyl) ethylenediamine solution (N113103-5 g, Aladdin) was then added to the mixture and incubated for another 5 min at room temperature. The absorbance was measured at 525 nm. The concentration of nitrite in the samples was calculated according to the standard curve prepared in parallel using sodium nitrite.

### 2.6 Quantitative PCR (q-PCR)

Total RNA was isolated from cells or blood samples using the *TransZol* UP RNA extraction reagent (ET111-01, TransGen Biotech). The RNA was converted to cDNA using Hifair^®^ III first Strand cDNA Synthesis SuperMix (11141ES60, Yeasen). After reverse transcription, PCRs were performed in an ABI 7900HT Real-Time PCR System (Applied Biosystems) with Hieff UNICON^®^ Power qPCR SYBR Green Master Mix (11197ES08, Yeasen). The PCR protocol involved denaturation at 95°C for 10 s and combined annealing and extension at 60°C for 30 s over 40 cycles. The melting curve was then generated to ensure that the amplification in each reaction was specific. *Actb* was used as the internal control. Fold changes in RNA levels were calculated using the ΔΔCt method. The PCR primers are listed in Supplementary Table S3 (Supplementary Material).

### 2.7 Western blot

Cells or tissue samples were washed with cold PBS twice and then lysed with cold radioimmunoprecipitation assay (RIPA) buffer (P0013B, Beyotime) supplemented with protease inhibitor cocktail (4693116001, Roche). Equal amounts (30 μg/lane) of denatured cell lysate were separated by SDS‒PAGE and then transferred to a polyvinylidene difluoride membrane (IPVH00010, Millipore). Target proteins on the membrane were probed with antibodies (see Reagents) and detected using the ImageQuant LAS 500 imager (GE Healthcare).

### 2.8 Whole blood LPS stimulation assay

Sixto eight-week-old male C57BL/6J mice were randomly divided into 3 groups and administered 0.5% CMC-Na vehicle, 100 mg/kg DMF (suspended in 0.5% CMC-Na), or 100 mg/kg MMF (dissolved in 0.5% CMC-Na) twice a day for 1 week. Blood was then drawn from retro-orbital veins into heparinized tubes and diluted with RPMI-1640 medium supplemented with 100 U/mL heparin at a ratio of 1:1 (v/v). The diluted blood was seeded into 48-well plates and treated *ex vivo* with LPS for 6 h. Afterwards, the supernatant was collected for ELISA, and the blood cells were collected for qPCR analysis.

### 2.9 Enzyme-linked immunosorbent assay (ELISA)

The levels of TNF-α and IL-6 in the samples were determined using the Mouse TNF ELISA Suit (555268, BD Biosciences) and Mouse IL-6 optEIA ELISA Suit (555240, BD Biosciences). The operations were conducted following the manufacturer’s instructions.

### 2.10 Ellman’s assay

GSH and test compounds were diluted with buffer A (400 mM Tris-HCl, pH = 7.4) to 2 mM and 200 μM, respectively, and warmed at 37°C. Equal volumes of prewarmed GSH and the test compounds were mixed in a tube and incubated at 37°C. An aliquot of 100 μL of the reaction mixture was sampled every 2 min and immediately added to 100 μL of 2 mM DTNB prepared in buffer B (buffer A with 1 mM EDTA) to stop the reaction. The absorbance was measured at 412 nm.

### 2.11 Reduced glutathione (GSH) and GSSG colorimetric assay

Cell samples were harvested and lysed by the snap freezing-thawing method. After deproteinization, the supernatant was collected for the determination of GSH and GSSG using the GSH and GSSG assay kit (S0053, Beyotime). The precipitate, which contained denatured proteins, was dissolved in 0.1 M NaOH, and the protein concentration was measured using the Pierce™ BCA protein assay kit (23227, Thermo Scientific). Finally, the amount of intracellular GSH or GSSG was normalized using the corresponding protein concentration.

### 2.12 Luciferase reporter assay

For the ARE-luciferase reporter assay, HEK293-ARE reporter cells were seeded into 96-well plates at a density of 5 × 10^4^ cells/mL and treated as indicated. Luciferase activity was detected using Steady-Glo^®^ Luciferase Assay Reagent (E2520, Promega) according to the manufacturer’s instructions.

For the dual-luciferase reporter assay, RAW264.7 cells were transfected with pGL4.13-Nfkbiz-promoter or pGL4.13-Nfkbiz-3UTR together with pRL-TK as the internal control for 24 h. Cells were then treated as indicated and harvested. The dual-luciferase activity was determined using the Duo-Lite Luciferase Assay Reagent (DD1205-01, Vazyme).

### 2.13 Chromatin immunoprecipitation (ChIP) assays

Chromatin for ChIP was prepared from RAW264.7 cells by fixing the cells in 1% formaldehyde for 10 min, followed by quenching with 125 mM glycine for 5 min. The cell pellet was resuspended and lysed with ChIP lysis buffer and sonicated to generate 200–1,000 bp DNA fragments with an ultrasonic sonicator. The antibodies used in this study for immunoprecipitation were rabbit monoclonal anti-STAT3 and anti-NF-κB antibodies. The precipitated DNA-chromatin products were purified, and the DNA levels were quantified by qPCR. The DNA levels are expressed as the percentage of input DNA.

### 2.14 Statistical analysis

Data are presented as the means ± S.E.M.s. The Shapiro‒Wilk normality test was used to assess whether data were normally distributed. Statistical analysis, as indicated in each figure legend, was performed using the GraphPad Prism 7.00 software (San Diego, CA, USA; RRID: SCR_000306). Differences at *p < 0.05* were considered statistically significant.

## 3 Results

### 3.1 Short-term exposure to DMF inhibited LPS-induced nitrite release by RAW264.7 cells

**FIGURE 1:**
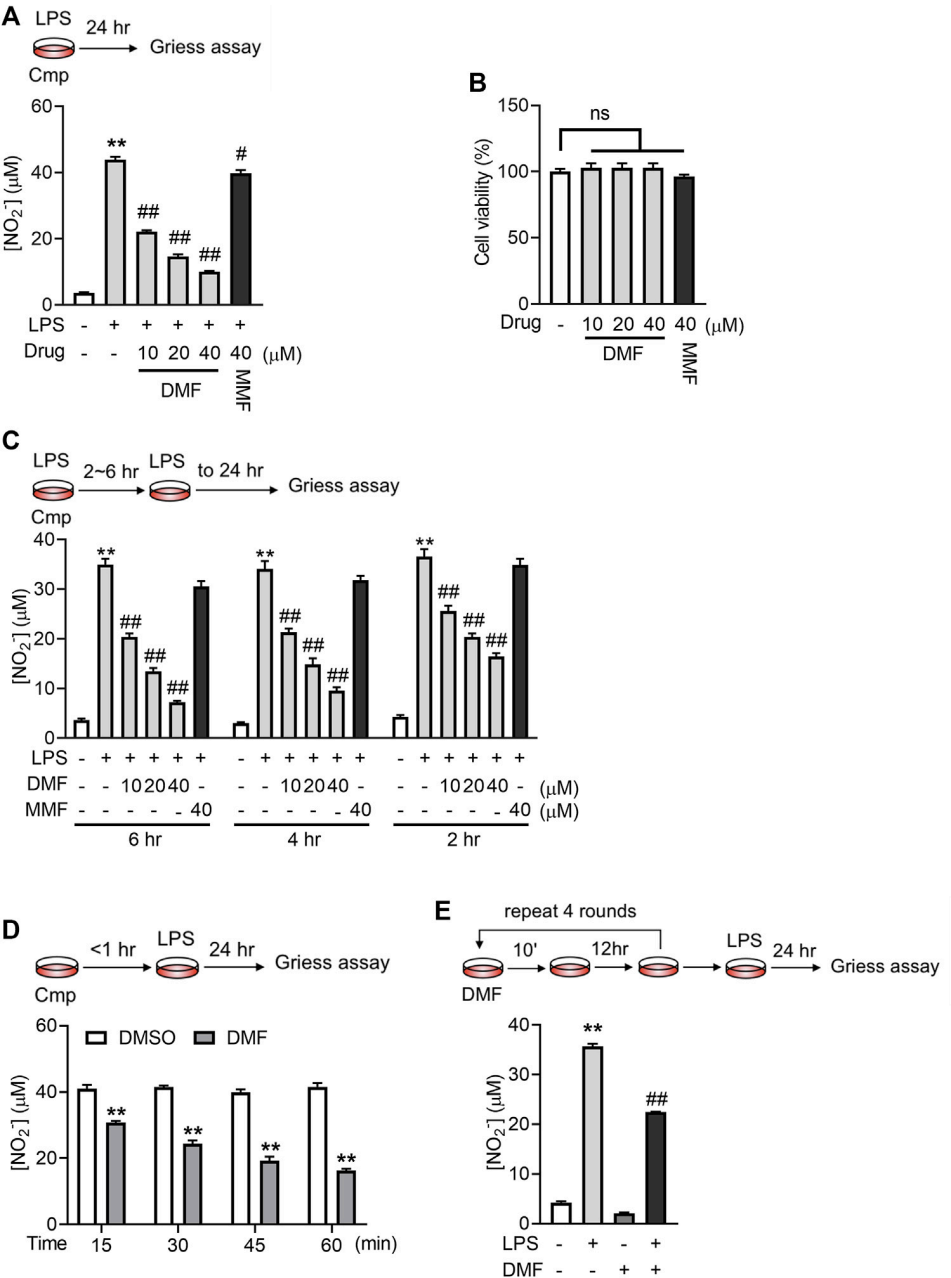
Short-term exposure to DMF inhibited LPS-mediated activation of RAW264.7 cells. **(A)** RAW264.7 cells were treated with 1 μg/mL LPS in the presence or absence of DMF/MMF for 24 h, and nitrite production in the indicated groups was quantified by the Griess assay (*n* = 3). **(B)** RAW264.7 cells were treated with DMF or MMF for 24 h, and the cell viability of the indicated groups was determined by MTT assay (*n* = 3). **(C)** RAW264.7 cells were treated with 1 μg/mL LPS in the presence or absence of DMF/MMF for 2–6 h and then cultured in medium with LPS only for a total of 24 h. The nitrite production from the indicated groups was quantified by the Griess assay (*n* = 3). **(D)** RAW264.7 cells were exposed to 40 μM DMF for 15–60 min and then cultured in medium with LPS only for 24 h. The nitrite production from the indicated groups was quantified by the Griess assay (*n* = 3). **(E)** Raw264.7 cells were transiently exposed to 10 μM DMF for 10 min and then rested in DMF-free medium for 12 h, followed by another round of transient DMF treatment. After four rounds of transient DMF treatment, cells were seeded into a 96-well plate, rested for 12 h, and challenged with 1 μg/mL LPS for 24 h. The nitrite production from the indicated groups was quantified by the Griess assay (*n* = 4). One-way ANOVA, **p < 0.01 vs. the untreated/DMSO group; ^#^ *p < 0.05*, ^##^ *p < 0.01* vs. the LPS group.

We first confirmed the effects of DMF on macrophage activation by coincubation of RAW264.7 cells with DMF and LPS. The nitrite level in the culture medium was taken as an indicator of RAW264.7-cell activation. As previously reported (To et al., 2015), DMF concentration-dependently inhibited LPS-induced nitrite release after 24 h treatment in RAW264.7 cells without affecting cell viability (Figures 1A, B). In contrast, its metabolite, MMF, demonstrated only minor effects (Figure 1A). When the coincubation time was shortened to within 6 h, only DMF was able to inhibit nitrite production (Figure 1C). Since the half-life of DMF in human serum was estimated to be 0.37 h (approximately 22 min) (Litjens et al., 2004b), the DMF incubation time was adjusted to the physiologically relevant exposure time. As shown in Figure 1D, DMF significantly suppressed the release of nitrite in RAW264.7 cells even when the incubation time was shortened to 15 min. In addition, significant inhibitory effects were also observed when cells were briefly exposed to lower concentrations of DMF for multiple times (Figure 1E), which mimics the long-term applications of DMF in patients. The above data showed that DMF but not MMF exhibits rapid inhibition on macrophages activation, suggesting that DMF may have distinctive anti-inflammatory effects.

### 3.2 Short-term exposure to DMF suppressed LPS-induced IκBζ expression in RAW264.7 cells

**FIGURE 2:**
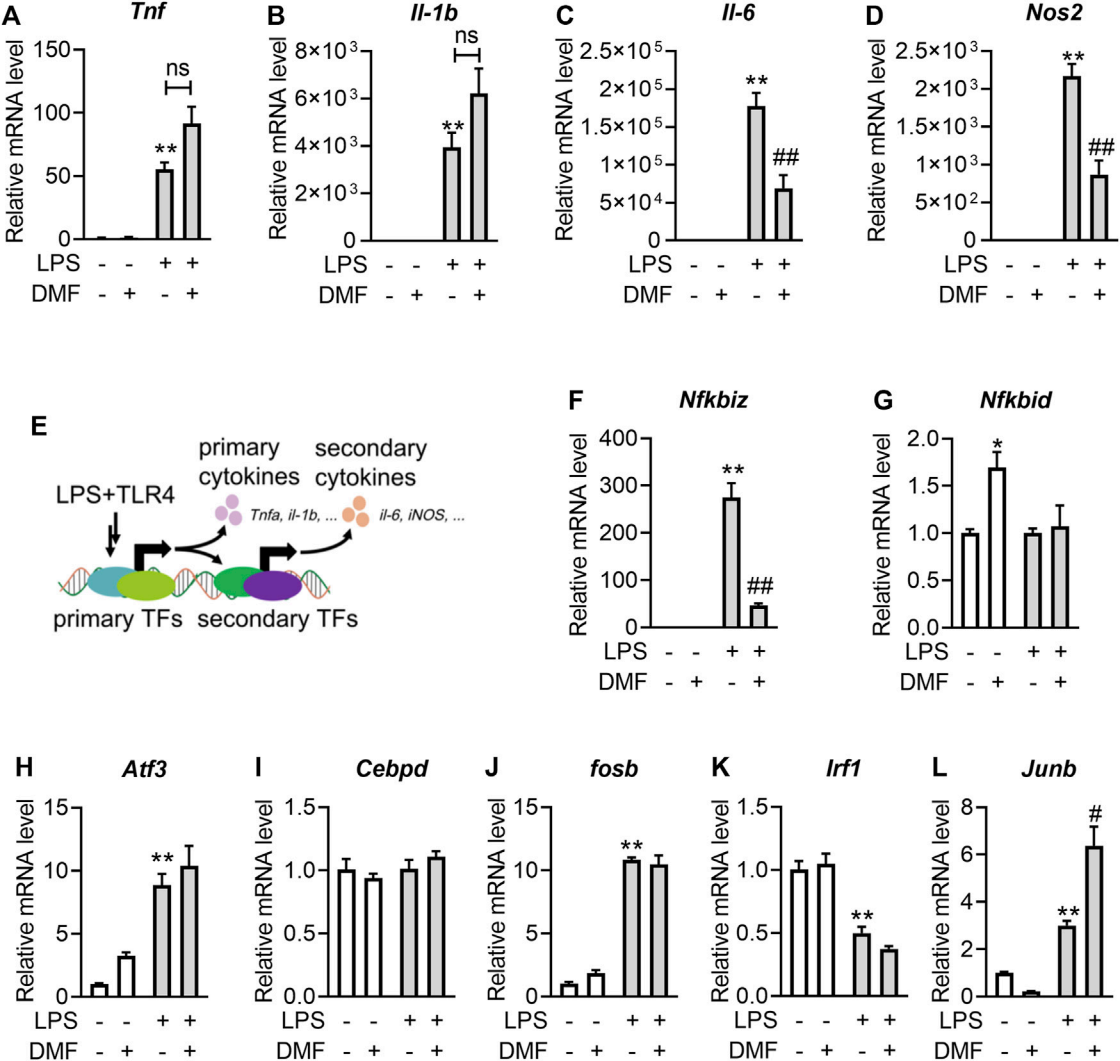
Short-term exposure to DMF suppressed the transcription level of IκBζ in RAW264.7 cells. **(A–D)** RAW264.7 cells were pretreated with 40 μM DMF for 1 h and then stimulated with 1 μg/mL LPS in the absence of DMF for 6 h. RT‒qPCR analysis of *Tnf* **(A)**, *Il-b* **(B)**, *Il-6* **(C)** and *Nos2* **(D)** mRNA expression in the indicated groups (*n* = 3). **(E)** Diagram of the primary and secondary transnational programs in the LPS/TRL4 pathway. **(F–L)** RAW264.7 cells were pretreated with 40 μM DMF for 1 h and then stimulated with 1 μg/mL LPS in the absence of DMF for 1 h. RT‒qPCR analysis of *Nfkbiz* **(F)**, *Nfkbid* **(G)**, *Atf3* **(H)**, *Cebpd* **(I)**, *Fosb* **(J)**, *Irf1* **(K)** and *Junb* **(L)** mRNA expression in the indicated groups (*n* = 3). **p < 0.01 vs. the untreated group by one-way ANOVA; ^##^ *p < 0.01* vs. the LPS group by one-way ANOVA; ns, not significant vs. the indicated group by *t*-test.

**FIGURE 3:**
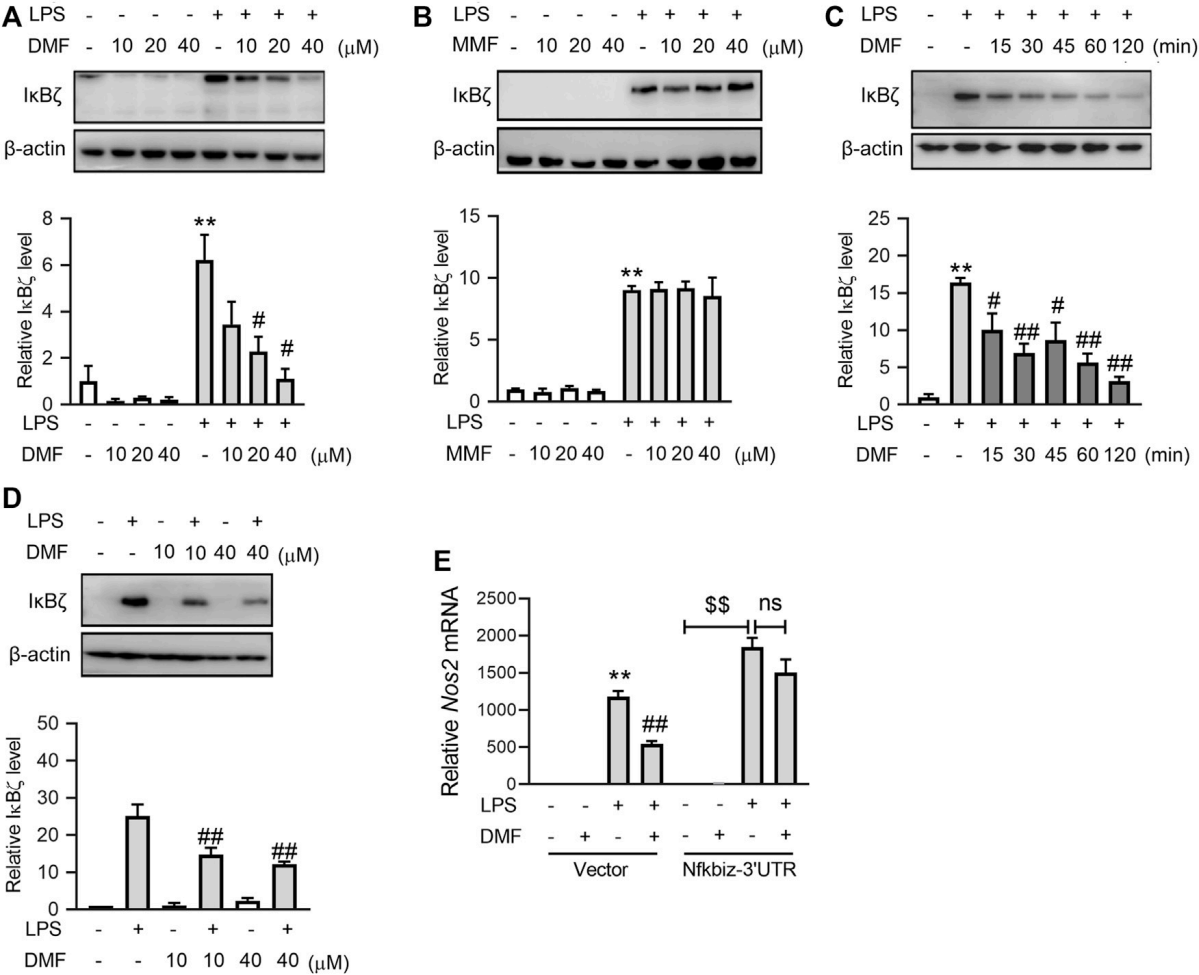
Short-term exposure to DMF suppressed the protein expression level of IκBζ in RAW264.7 cells. **(A,B)** RAW264.7 cells were pretreated with the indicated concentrations of DMF **(A)** or MMF **(B)** for 1 h and then stimulated with 1 μg/mL LPS in the absence of DMF for 1 h. Western blot analysis and quantification of IκBζ from the indicated groups (*n* = 3). **(C)** RAW264.7 cells were pretreated with 40 μM DMF for the indicated time and then stimulated with 1 μg/mL LPS in the absence of DMF for 1 h. Western blot analysis and quantification of IκBζ from the indicated groups (*n* = 4). **(D)** RAW264.7 cells were transiently treated with DMF as indicated (Figure 1) and then stimulated with 1 μg/mL LPS for 1 h. Western blot analysis and quantification of IκBζ from the indicated groups (*n* = 3). **(E)** pSV40 or pSV40-Nfkbiz-3′UTR plasmid-transfected cells were pretreated with 40 μM DMF for 1 h and then stimulated with 1 μg/mL LPS in the absence of DMF for 6 h. RT‒qPCR analysis of *Nos2* mRNA expression in the indicated groups (*n* = 4)*.* *p < 0.05, **p < 0.01 vs. the untreated group by one-way ANOVA; ^#^ *p < 0.05*, ^##^ *p < 0.01* vs. the LPS group by one-way ANOVA; ^$$^ *p < 0.01* vs. the indicated group by *t*-test; ns, not significant vs. the indicated group by *t*-test.

To explore the inhibitory effect of DMF on inflammation in greater detail, the transcription levels of proinflammatory genes, including *Tnf*, *Il-1b*, *Il-6*, and *Nos2* (Figures 2A–D)*,* were measured. Interestingly, short-term exposure to DMF significantly inhibited the transcription of *Il-6* (Figure 2C) and *Nos2* (Figure 2D) but not *Tnf* (Figure 2A) or *Il-1b* (Figure 2B). Considering that both *Il-6* and *Nos2* transcription belong to the secondary transcription program in the LPS/TLR4 pathway (Figure 2E) (Medzhitov and Horng, 2009; Ramirez-Carrozzi et al., 2009), the mRNA levels of transcription factors (TFs) that regulate the secondary transcription program, including *Fosb*, *Nfkbid*, *Cebpd*, *Nfkbiz*, *Atf3*, *Junb*, and *Irf1*(Figures 2F–L), were detected. Among these TFs, DMF inhibited the transcription of *Nfkbiz* mRNA to the greatest extent (Figure 2F). Additionally, western blotting confirmed that DMF inhibited the expression of IκBζ (the protein product of the *Nfkbiz* gene) in a dose-dependent manner (Figure 3A); however, the metabolite MMF did not show similar effects (Figure 3B). The inhibitory effect of DMF on IκBζ expression was also observed when the exposure time was shortened to 15 min (Figure 3C). Transient exposure to DMF multiple times also inhibited LPS-induced expression of IκBζ (Figure 3D). Importantly, in LPS-treated RAW264.7 cells with ectopic IκBζ expression, DMF failed to inhibit the transcription level of *Nos2*, further confirming that IκBζ is the downstream effector in the anti-inflammatory action of DMF (Figure 3E). Taken together, these data indicate that IκBζ may be a key target underlying the rapid anti-inflammatory effects of DMF.

### 3.3 Oral DMF suppressed the LPS-induced inflammatory response in a whole-blood assay

**FIGURE 4:**
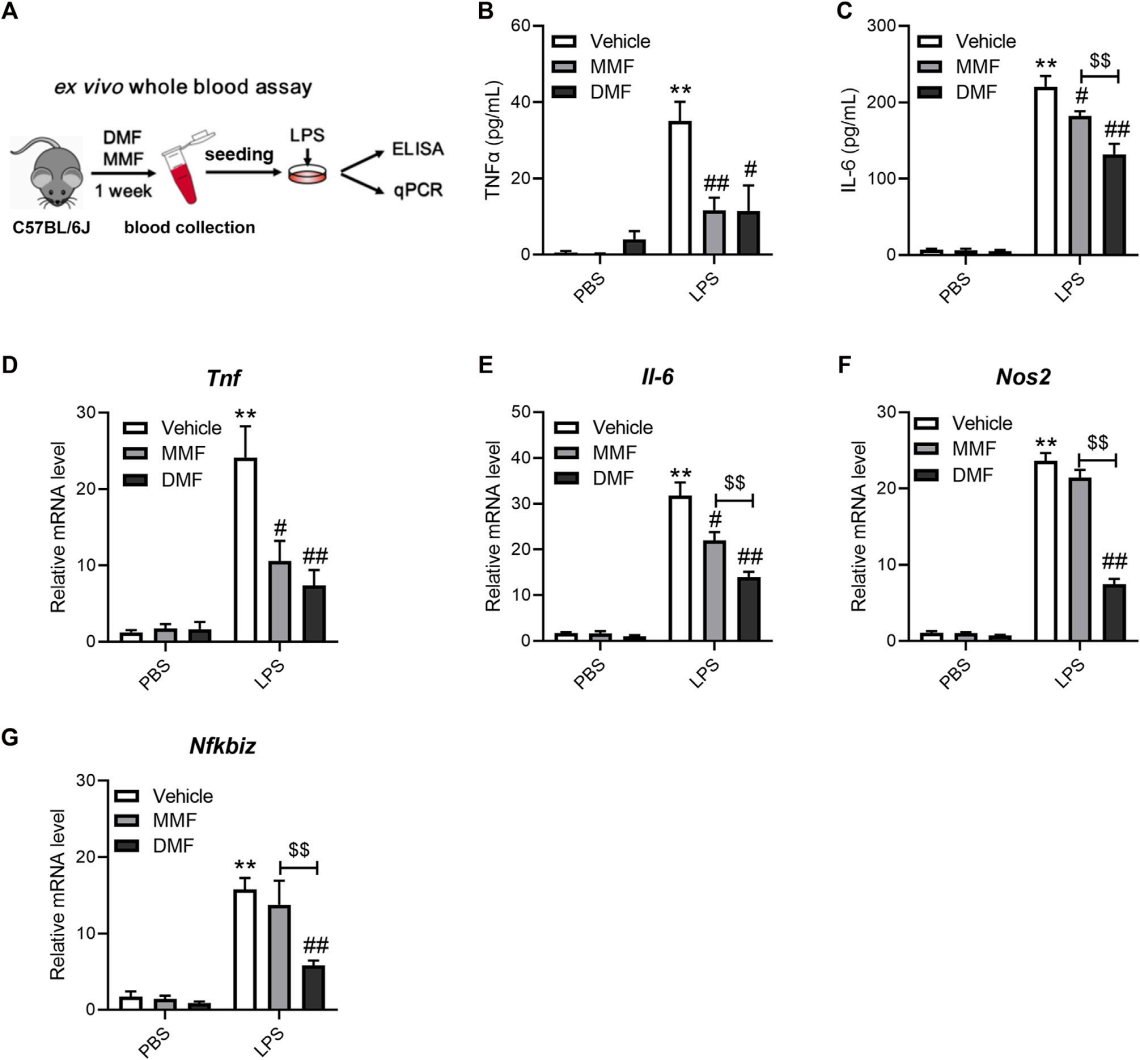
Oral administration of DMF exhibits distinct anti-inflammatory effects in *ex vivo* whole blood LPS stimulation assays. **(A)** Diagram of the study design. Mice were orally administered DMF or MMF for 1 week, and blood samples were collected for *ex vivo* whole blood assays. **(B,C)** Peripheral blood cells stimulated with 1 μg/mL LPS for 6 h *ex vivo.* ELISA analysis of TNFα **(B)** and IL-6 **(C)** in the indicated groups (*n* = 7). **(D–G)** Peripheral blood cells stimulated with 1 μg/mL LPS for 1 h. RT‒qPCR analysis of *Tnf* **(D)**, *Il-6* **(E)**, *Nos2* **(F)** and *Nfkbiz* **(G)** mRNA expression in the indicated groups (*n* = 6–7). **p < 0.01 vs. the untreated group by one-way ANOVA; ^#^ *p < 0.05*, ^##^ *p < 0.01* vs. the LPS group by one-way ANOVA; ^$$^ *p < 0.01* vs. the indicated group by *t*-test.

If DMF indeed has a direct impact on immune cells *in vivo*, the anti-inflammatory effects of DMF should be distinct from those of its metabolite, MMF, in mice. The above results show that DMF, but not MMF, strongly inhibited LPS-induced macrophage activation *in vitro*. We next employed a whole-blood assay comparing the effects of DMF and MMF on inflammation *in vivo* to test the idea above (Figure 4A). Mice were orally administered the same dose of DMF or MMF for 7 consecutive days. Then, blood samples were collected, and their reactivity to LPS stimulation was profiled *ex vivo*. The whole-blood assay revealed that blood cells from mice treated with DMF or MMF produced decreased levels of TNFα and IL-6 after LPS stimulation (Figures 4B, C). The transcription of *Tnf* and *Il6* mRNA was also decreased in both groups (Figures 4D, E). However, it should be noted that in comparison with MMF, DMF exerted a more profound suppressive effect on the production of IL-6 at both the protein and mRNA levels (Figures 4C, E). Notably, the transcription of *Nos2* and *Nfkbiz* were both inhibited by oral DMF but not by oral MMF (Figures 4F, G), which were consistent with the *in vitro* results. These results revealed that the anti-inflammatory effects of DMF and MMF differ in mice, suggesting that MMF dose not account for all of the effects of DMF *in vivo*.

### 3.4 DMF inhibits LPS-induced IκBζ expression by GSH depletion

**FIGURE 5:**
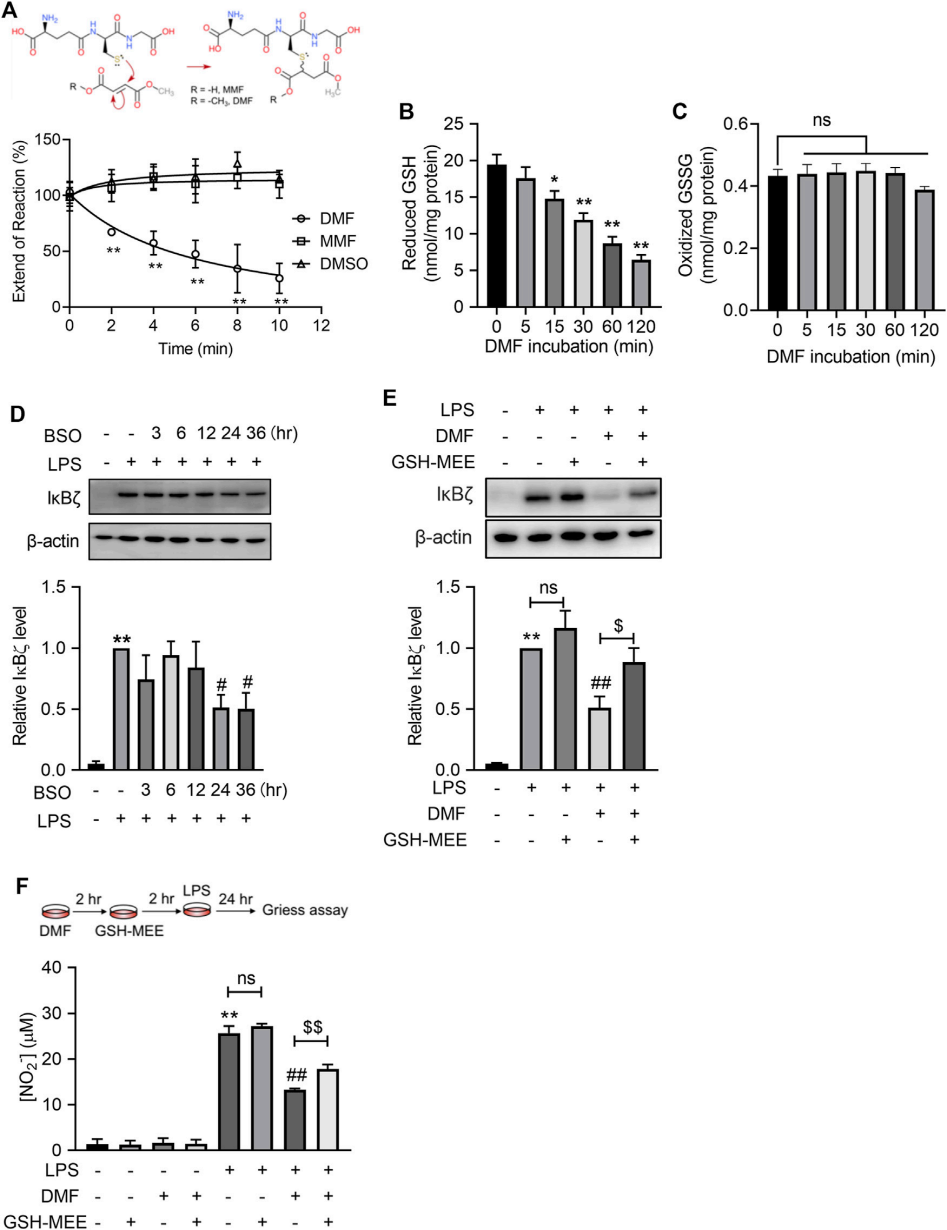
DMF suppressed LPS-induced IκBζ expression by rapid GSH depletion. **(A)** DMF or MMF (100 μM) was incubated with 1 mM GSH under physiological conditions (37°C, pH = 7.4) and then monitored by Ellman’s assay (*n* = 4). **(B,C)** RAW264.7 cells were treated with 40 μM DMF for the indicated time and were then harvested for the determination of intracellular GSH **(B)** and GSSG **(C)**. **(D)** RAW264.7 cells were treated with 100 μM BSO for the indicated times. Western blot analysis and quantification of IκBζ in the indicated groups (*n* = 3). **(E)** RAW264.7 cells were treated with 40 μM DMF for 2 h and then supplied with 2 mM GSH-MEE in the absence of DMF, followed by 1 h LPS stimulation in the absence of either GSH-MEE or DMF. Western blot analysis and quantification of IκBζ in the indicated groups (*n* = 4). **(F)** RAW264.7 cells were treated with 40 μM DMF for 2 h and then supplied with 2 mM GSH-MEE in the absence of DMF, followed by a 24 h LPS stimulation in the absence of either GSH-MEE or DMF. The nitrite production from the indicated groups was quantified by the Griess assay (*n* = 3). *p < 0.05, **p < 0.01 vs. the untreated group by one-way ANOVA; ^##^ *p < 0.01* vs. the LPS group by one-way ANOVA; ^$^ *p < 0.05*, ^$$^ *p < 0.01* vs. the indicated group by *t*-test; ns, not significant vs. the indicated group by *t*-test.

We next attempted to explore the mechanisms underlying DMF inhibition on LPS-induced IκBζ expression. Both DMF and MMF are prone to react with nucleophiles through Michael addition, based on their structures containing α,β-unsaturated carbonyls (Figure 5A). GSH is one of the most abundant nucleophiles within cells and plays an important role in regulating the proinflammatory activation of macrophages (Buchmuller-Rouiller et al., 1995; Muri and Kopf, 2021). However, under physiological conditions (pH 7.4°C and 37°C), DMF, but not MMF, extensively reacted with GSH within minutes (Figure 5A). Additionally, DMF quickly depleted intracellular GSH without increasing the oxidized GSSG (Figures 5B, C). We hypothesized that rapid GSH depletion by DMF might account for its quick inhibition of LPS-induced IκBζ expression. To test this idea, the GSH synthesis inhibitor buthionine sulfoximine (BSO) was applied to mimic the GSH-depleting effect of DMF. Notably, BSO suppressed the expression of IκBζ (Figure 5D). In addition, glutathione monoethyl ester (GSH-MEE), a membrane-permeable GSH derivative, was supplied to DMF-treated cells to restore the intracellular GSH pool. GSH-MEE supplementation increased nitrite production and restored IκBζ expression in DMF-treated cells (Figures 5E, F). Taken together, these results confirm the regulatory role of GSH in IκBζ expression and indicate that DMF may inhibit IκBζ expression through GSH depletion.

### 3.5 DMF suppresses IκBζ expression through Nrf2 activation

**FIGURE 6:**
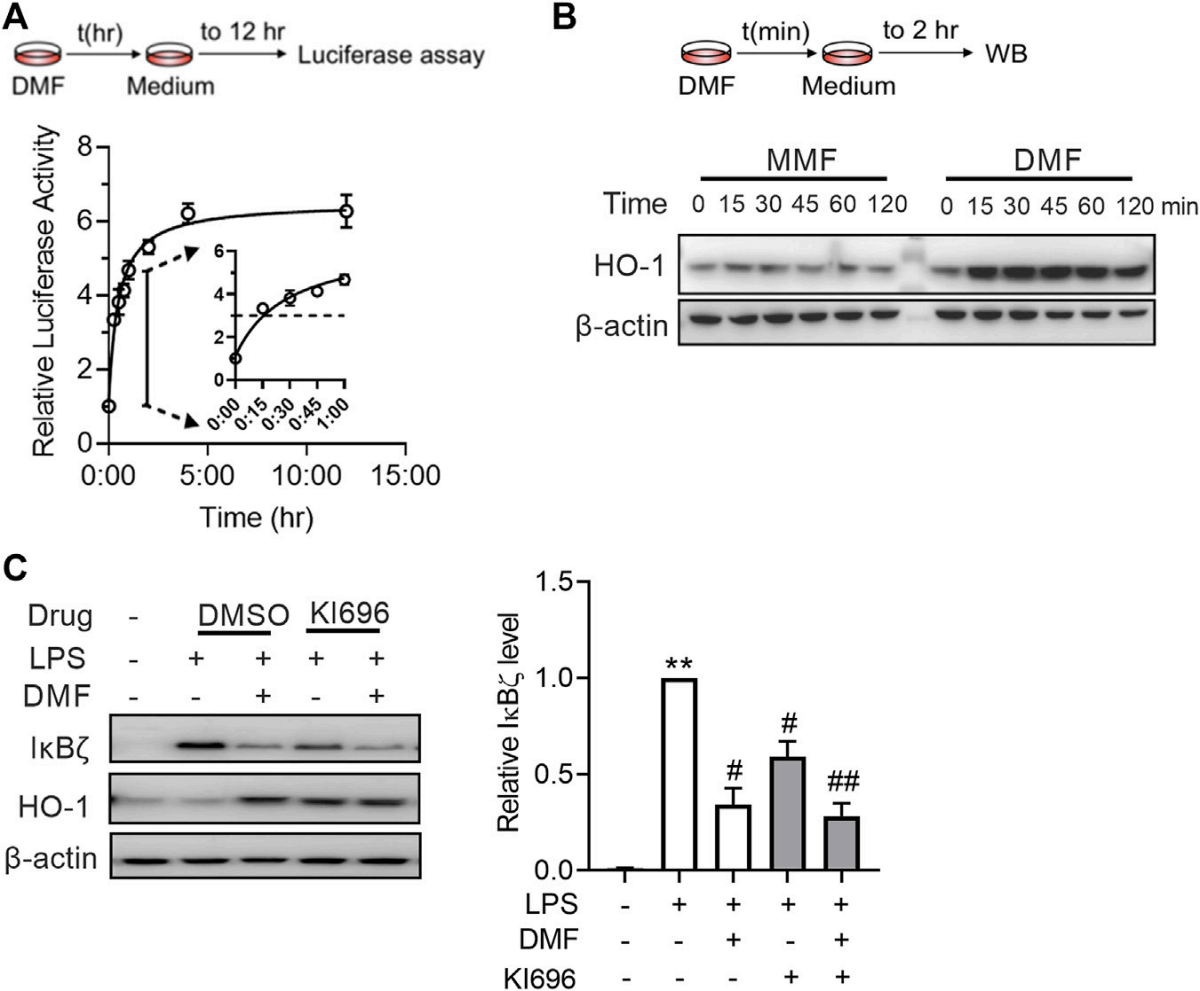
DMF inhibited LPS-induced IκBζ expression by rapid Nrf2 activation. **(A)** HEK293-ARE reporter cells were treated with 40 μM DMF for the indicated time and then cultured in DMF-free medium for a total of 12 h. A luciferase assay was applied to evaluate Nrf2 activation (*n* = 3). **(B)** RAW264.7 cells were treated with 40 μM DMF for the indicated time and then cultured in DMF-free medium for 2 h. Western blot analysis of HO-1 from the indicated groups. **(C)** RAW264.7 cells were treated with 10 μM KI696 for 24 h and then 40 μM DMF for 1 h, followed by a 1-h LPS stimulation. Western blot analysis and quantification of IκBζ in the indicated groups (*n* = 3). **p < 0.01 vs. the untreated group by one-way ANOVA; ^#^ *p < 0.05*, ^##^ *p < 0.01* vs. the LPS group by one-way ANOVA.

A As DMF has been characterized as a potent covalent Nrf2 activator, we wondered whether short-term exposure to DMF is enough to activate the Nrf2 pathway. Using the luciferase reporter assay, we observed that 12 h of exposure to DMF induced maximal (approximately 6-fold) luciferase signal enhancement in cells stably expressing the NRF2/ARE-responsive luciferase reporter gene (Figure 5A). Although the intensity of the luciferase signal decreased with shorter exposure times, 15 min of exposure to DMF still resulted in half-maximal (∼3-fold) signal enhancement (Figure 6A), indicating that short-term exposure to DMF is sufficient to activate the Nrf2 pathway. Activation of the Nrf2 pathway by short-time exposure to DMF was further confirmed by western blot, as indicated by the enhanced expression of HO-1 in cells (Figure 6B). However, short-term exposure to the metabolite MMF failed to activate the Nrf2 pathway, further suggesting that the agonistic effect of DMF on the Nrf2 pathway is one of the distinctive mechanisms by which it exerts anti-inflammatory effects.

Next, we investigated whether Nrf2 plays a role in the inhibitory effect of DMF on IκBζ expression. To test this notion, RAW264.7 cells were treated with KI696, a non-covalent Nrf2 activator that activates Nrf2 through a mechanism different from that of DMF (Davies et al., 2016). KI696 activated the expression of HO-1 in RAW264.7 cells and inhibited the expression of IκBζ in LPS-treated RAW264.7 cells (Figure 6C). Taken together, the above data suggest that Nrf2 activation is also a mechanism by which DMF inhibits IκBζ expression.

### 3.6 Nrf2 knockout failed to abolish the inhibitory effect of DMF on IκBζ

**FIGURE 7:**
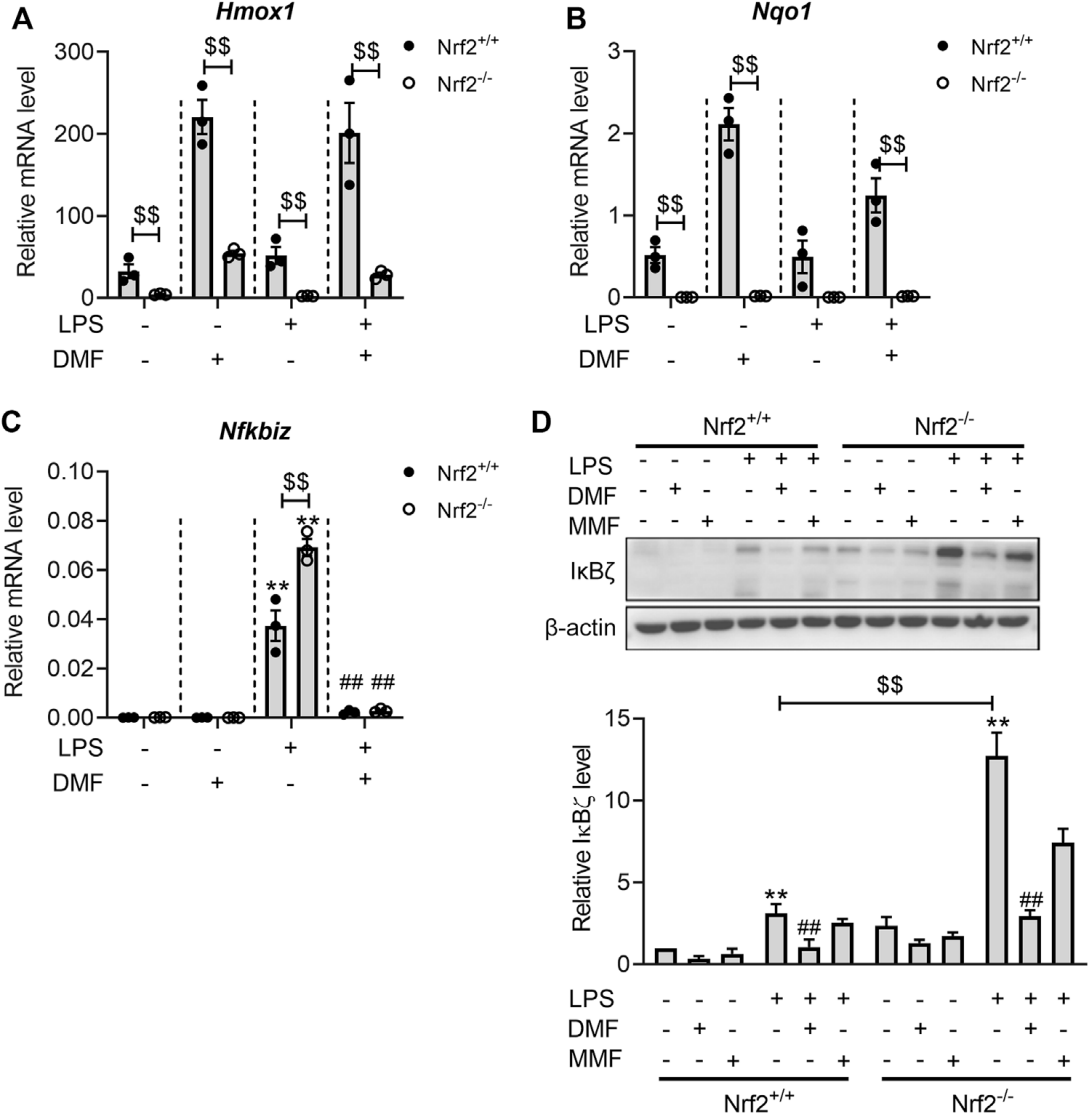
DMF inhibited LPS-induced IκBζ expression in both Nrf2^+/+^ and Nrf2^−/−^ BMDMs. **(A,B)** Nrf2^+/+^ or Nrf2^−/−^ BMDMs were treated with 40 μM DMF/MMF for 1 h and then stimulated with 1 μg/mL LPS in the absence of DMF/MMF for 6 h. RT‒qPCR analysis of *Hmox1* **(A)** and *Nqo1* **(B)** mRNA expression in the indicated groups (*n* = 3). **(C,D)** Nrf2^+/+^ or Nrf2^−/−^ BMDMs were treated with 40 μM DMF/MMF for 1 h and then stimulated with 1 μg/mL LPS in the absence of DMF/MMF for 1 h **(C)** RT‒qPCR analysis of *Nfkbiz* mRNA expression in the indicated groups (*n* = 3). **(D)** western blot analysis and quantification of the IκBζ expression level (*n* = 3). **p < 0.01 vs. the untreated group by one-way ANOVA. ^##^ *p < 0.01* vs. the LPS group by one-way ANOVA. ^$$^ *p < 0.01* vs. the Nrf2^−/−^ group by *t*-test.

To investigate whether DMF is likely to regulate IκBζ expression beyond Nrf2 activation, the effects of DMF were examined in both Nrf2^+/+^ and Nrf2^−/−^ BMDMs. The lack of functional Nrf2 in the Nrf2^−/−^ BMDMs was confirmed by the lower levels of *Hmox1* and *Nqo1* mRNA in both resting and LPS-activated states (Figures 7A, B). Consistent with the results in RAW264.7 cells, LPS stimulation significantly upregulated both the mRNA and protein levels of IκBζ in Nrf2^+/+^ and Nrf2^−/−^ BMDMs (Figures 7C, D). In Nrf2^−/−^ BMDMs, there was a higher level of IκBζ than in Nrf2^+/+^ BMDMs (Figures 7C, D), further validating the regulatory role of Nrf2 in IκBζ expression. Importantly, DMF also significantly suppressed IκBζ expression in the Nrf2^−/−^ BMDMs (Figures 7C, D), whereas the metabolite MMF exerted little impact on IκBζ expression in both the Nrf2^−/−^ and Nrf2^+/+^ BMDMs (Figure 7D). The data above suggest that the mechanisms by which DMF inhibits IκBζ may involve pathways other than Nrf2.

### 3.7 DMF inhibits IκBζ expression through the STAT3 pathway

**FIGURE 8:**
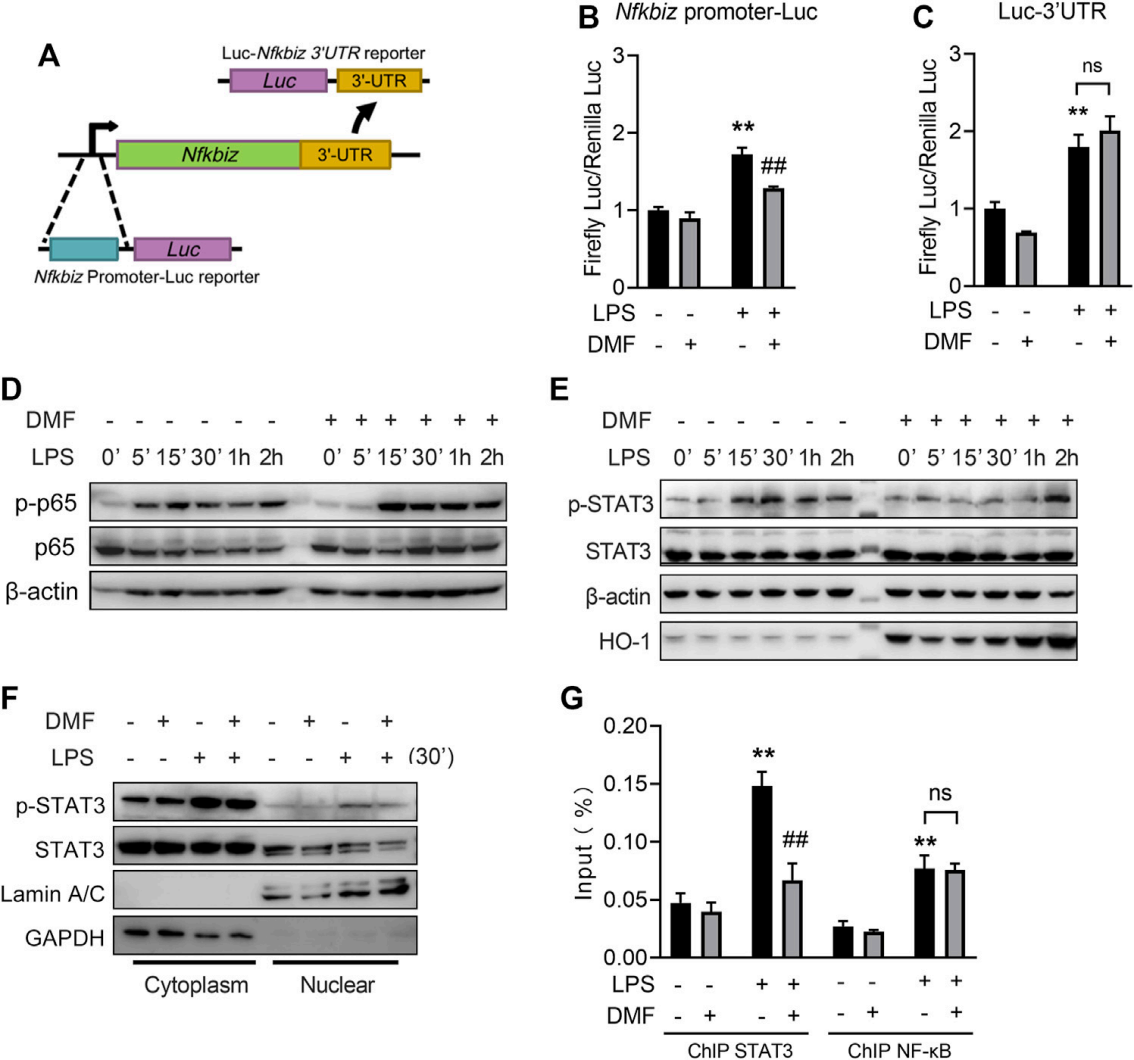
DMF inhibits IκBζ expression through the STAT3 pathway. **(A)** Construction of the Nfkbiz-promoter-Luc reporter and the Luc-Nfkbiz-3UTR reporter. **(B)** Cells transiently transfected with pGL4.13-Nfkbiz-promoter or pRL-TK were treated with 40 μM DMF for 1 h and then stimulated with 1 μg/mL LPS for 6 h, followed by the dual luciferase assay (*n* = 3). **(C)** Cells transiently transfected with pGL4.13-Nfkbiz-3UTR or pRL-TK were treated with 40 μM DMF for 1 h and then stimulated with 1 μg/mL LPS for 6 h, followed by the dual luciferase assay (*n* = 3). **(D,E)** Cells were treated with 40 μM DMF for 1 h and then stimulated with 1 μg/mL LPS in the absence of DMF for the indicated time. Western blot analysis of the indicated proteins. **(F)** Cells were treated with 40 μM DMF for 1 h and then stimulated with 1 μg/mL LPS in the absence of DMF for 30 min. The cytoplasmic and nuclear fractions were isolated for western blot analysis. **(G)** Cells were treated with 40 μM DMF for 1 h and then stimulated with 1 μg/mL LPS for 6 h. ChIP‒qPCR analyses were performed using STAT3 **(G)** or NF-κB antibody **(H)** (*n* = 3). **p < 0.01 vs. the untreated group by one-way ANOVA. ^##^ *p < 0.01* vs. the LPS group by one-way ANOVA; ns, not significant vs. the indicated group by *t*-test.

The full induction of IκBζ expression requires both the activation of its promoter and the stabilization of its mRNA (Muromoto et al., 2019). To investigate the mechanism of DMF-regulated IκBζ inhibition, the promoter region of the *Nfkbiz* gene and the 3′-UTR elements that govern the stability of *Nfkbi*z mRNA were introduced into luciferase reporter constructs (Figure 8A). In the Nfkbiz-promoter-luciferase assay, DMF-treated cells showed a decreased luciferase signal upon LPS stimulation (Figure 8B). In the luciferase-3′-TUR assay, DMF failed to suppress the luciferase signal induced by LPS stimulation (Figure 8C). These results revealed that DMF suppresses the promoter activity of *Nfkbiz* but not the stability of its mRNA.

It has been reported that NF-κB and STAT3 govern *Nfkbi*z promoter activity (Muller et al., 2018; Muromoto et al., 2019). For NF-κB, DMF delayed the early phosphorylation of p65 after 5 min of LPS stimulation but did not affect later phosphorylation events (Figure 8D). DMF-treated cells exhibited delayed STAT3 phosphorylation, a hallmark of STAT3 protein activation, in response to LPS challenge (Figure 8E). Moreover, DMF also reduced the nuclear translocation of phosphorylated STAT3 (Figure 8F). To further investigate the role of DMF in regulating *Nfkbiz* expression, we performed ChIP‒qPCR assays using anti-STAT3 or anti-NF-κB Ab. Markedly increased binding to the *Nfkbiz* promoter was observed for STAT3 and NF-κB in the cell response to LPS treatment. However, DMF only significantly abrogated the binding of constitutive STAT3, but not NF-κB, to the IκB-ζ promoter region (Figure 8G).

Together, the results mentioned above indicate that STAT3 signalling is involved in DMF-mediated *Nfkbiz* transcription inhibition.

## 4 Discussion

**FIGURE 9:**
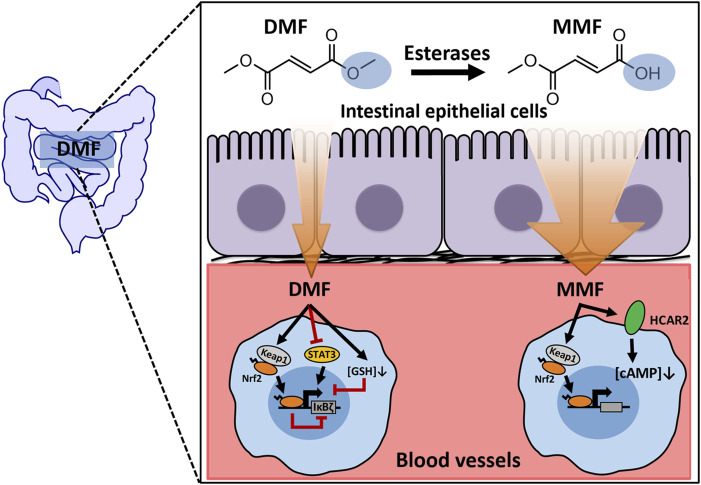
A working model depicting the mechanisms of IκBζ expression regulated by short-term exposure to DMF.

At present, the exact pharmacological activity of DMF in treating psoriasis and MS remains unclear. Due to metabolic instability and high chemical reactivity, DMF is present in the blood for a short time (Litjens et al., 2004b). Whether DMF has a direct therapeutic role remains unknown. Previous studies have neglected this key issue, and conclusions based on long-term DMF treatment *in vitro* should thus be interpreted with caution. Our study first took this issue into account and focused on the effect of short-term exposure to DMF to provide more insights regarding the role of parent DMF *in vivo* (Figure 9).

The structure of DMF is very simple and has a relatively small size in comparison to other therapeutic compounds on the market. The esterification of carboxyl groups on both sides renders DMF electrically neutral. These molecular properties facilitate the ability of DMF to permeate cell membranes. The metabolic product GS-DMS appears in portal vein blood within 2 minutes after gastrointestinal administration of DMF (Dibbert et al., 2013). In addition, in this study, short-term (15 min) treatment with DMF was found to significantly inhibit nitrite release and IκBζ expression in RAW264.7 cells. These phenomena support the excellent membrane-permeating ability of DMF.

Even though DMF may rapidly travel across the gastrointestinal barrier and enter the blood circulation, the detection of DMF in the blood using routine analytical techniques remains challenging because of the instability and poor ionization efficiency of this molecule (Junnotula and Licea-Perez, 2016). Therefore, obtaining direct evidence of the presence of DMF in the blood is difficult. However, comparing the *in vivo* efficacy of DMF and its metabolite MMF might help determine whether DMF plays a functional role *in vivo*. In this study, DMF and MMF were found to induce some common responses, but some of their effects also clearly differed, including the inhibition of *Nos2* and *Nfkbiz* transcription induced by LPS in blood cells. These observations suggested that the efficacy of DMF and MMF is not identical, and regulation of Nfkbiz expression may be the key step underlying functional differences between the two molecules.

Encoded by the *Nfkbiz* gene, IκBζ is an atypical member of the nuclear IκB family. Distinct from cytoplasmic IκB proteins, IκBζ possesses a transactivating domain and targets the expression of proinflammatory genes in collaboration with NF-κB (Yamazaki et al., 2001; Trinh et al., 2008). Recently, IκBζ was indicated to play a crucial role in the antipsoriatic effects mediated by anti-IL-17A treatment (Bertelsen et al., 2020). Moreover, IκBζ knockout mice showed a defect in Th17-cell development and were thus resistant to EAE (Okamoto et al., 2010), a preclinical model of human MS. These studies suggest that IκBζ might become a therapeutic target for psoriasis and MS.

In this study, DMF was found to strongly inhibit LPS-induced expression of IκBζ among a panel of TFs in RAW264.7 cells. More importantly, this inhibition was achieved even when DMF was applied for a short time. The quick inhibition of IκBζ expression by DMF may account for the reduction in nitrite release by RAW264.7 cells shortly after DMF treatment because the ectopic expression of IκBζ restored the *Nos2* mRNA level in RAW264.7 cells. Moreover, a recent study also reported that DMF compromised IL-17-induced expression of IκBζ in human keratinocytes (Ohgakiuchi et al., 2020). Thus, inhibiting IκBζ expression under proinflammatory conditions is one of the therapeutic mechanisms of DMF in psoriasis and MS.

Further investigation revealed that DMF inhibits IκBζ expression through multiple mechanisms, including GSH depletion, Nrf2 activation, and STAT3 phosphorylation inhibition. The multitarget activity of DMF should result from its unique chemical structure. The key functional groups in DMF are symmetric α,β-unsaturated carbonyls, which afford DMF high electrophilicity towards the thiol groups of biological targets (Schmidt et al., 2007; Zhang et al., 2021). GSH is a major thiol donor within cells and regulates immune responses by maintaining intracellular redox homeostasis (Muri and Kopf, 2021). In this study, GSH was found to regulate the expression of IκBζ in macrophages. DMF rapidly depleted intracellular GSH and thus disrupted redox homeostasis within macrophages, which possibly hampered the signal transduction process. More investigation is needed to further examine this process in molecular detail.

Interestingly, we found that DMF did not increase the oxidized GSSG while deplete the reduced GSH. The phenomenon that the amount of GSSG remained steady during DMF treatment indicated that the cells did not timely replenish GSH by recycling GSSG. Without timely regeneration of GSH, DMF is thereby able to react with cysteine residues of proteins. The reactivity of a cysteine residue is affected by nearby metal ions or basic amino acid residues, both of which promote deprotonation of the thiol group (Poole, 2015). Due to the presence of multiple basic amino acid residues in close proximity, Cys151 of Keap1 is highly reactive and a preferred target of electrophilic compounds, including DMF (Brennan et al., 2015; Suzuki et al., 2019; Dayalan Naidu and Dinkova-Kostova, 2020). The binding of DMF to Cys151 results in the inhibition of Keap1 and thereby the activation of Nrf2-ARE signalling. In this study, we found that DMF quickly promoted Nrf2 activation in a manner that resembled its ability to deplete GSH, indicating that DMF simultaneously acts on GSH and protein cysteine residues upon its entrance into cells. Notably, multiple pieces of evidence from chemical Nrf2 activation, Nrf2 overexpression, and Nrf2 knockout experiments suggested that Nrf2 negatively regulates LPS-induced IκBζ expression. To the best of our knowledge, this is the first study demonstrating the connection between Nrf2 and IκBζ, which adds new insights into understanding how Nrf2 activation constrains inflammatory responses. However, how Nrf2 downregulates IκBζ is still unclear and should be the focus of future exploration.

The results from Nrf2^−/−^ BMDMs revealed that there is a mechanism underlying DMF-mediated IκBζ inhibition in addition to Nrf2 activation. Using a luciferase assay, we first confirmed that DMF suppresses the promoter activity of *Nfkbiz*. Then, we found that DMF inhibits the phosphorylation of STAT3 and reduces its translocation into the nucleus. Phosphorylated STAT3 can recruit Sp1 and p65 to form transcription complexes in the nucleus and thus promote inflammatory gene transcription (Bharadwaj et al., 2020). The ChIP assay revealed that DMF abrogates the binding of STAT3 to the *Nfkbiz* promoter, leading to the suppression of *Nfkbiz* transcription.

In summary, combined with previous studies, our data suggest that circulating parent DMF molecules could have a direct impact on the immune system, which might generate additional therapeutic benefits in addition to those of its metabolite, MMF. This additional pharmacological activity of DMF has not been validated in humans. Notably, MMF was recently approved by the FDA as a monotherapy for MS based on the similar pharmacokinetic profiles of MMF and DMF (Berger et al., 2021). Real-world data will disclose whether these two drugs are truly bioequivalent, which will help determine whether DMF contributes to the overall therapeutic efficacy in humans and the importance of this mechanism.

## 5 Conclusion

This study demonstrates the rapid anti-inflammatory effects of DMF both *in vitro* and *in vivo*. Short-term exposure to DMF inhibits LPS-induced IκBζ expression in macrophages through multiple mechanisms, including the depletion of GSH, activation of Nrf2 and inhibition of constitutive STAT3 binding to the IκBζ promoter. These findings support the idea that the parent DMF is likely to have a direct impact on immune cells *in vivo* before being hydrolysed to MMF, providing key evidence for the direct therapeutic effect of DMF.

## Data Availability

The raw data supporting the conclusion of this article will be made available by the authors, without undue reservation.
