# Supplementary material for: Short-term exposure to dimethyl fumarate (DMF) inhibits LPS-induced IκBζ expression in macrophages
Source: Front Pharmacol. 2023 Feb 1;14:1114897. doi: 10.3389/fphar.2023.1114897 (PMC9929133; doi:10.3389/fphar.2023.1114897)
Supplement: Supplementary file 1 [file Table1.DOCX]

Table S1. Antibodies used in this study

| Antibody | Experiment | Dilution | Source | Cat# |
| --- | --- | --- | --- | --- |
| Rabbit anti-NFKBIZ pAb | WB | 1:1000 | Proteintech | 14014-1-AP |
| Mouse anti-β actin mAb | WB | 1:2000 | ZSGB-Bio | TA-09 |
| Rabbit anti-HO-1 mAb | WB | 1:1000 | Abcam | ab52947 |
| Rabbit anti-NQO1 mAb | WB | 1:1000 | Abcam | ab80588 |
| Rabbit anti-p-p65 (S536) mAb | WB | 1:1000 | Cell Signaling Technology | 3033T |
| Rabbit anti-p65 pAb | WB | 1:1000 | Proteintech | 10745-1-AP |
| Rabbit anti-p-STAT3(Y705) mAb | WB | 1:1000 | Cell Signaling Technology | 9145T |
| Rabbit anti-p-STAT3(S727) mAb | WB | 1:1000 | Cell Signaling Technology | 9134T |
| Mouse anti-STAT3 mAb | WB | 1:1000 | Cell Signaling Technology | 9139T |
| HRP-labeled goat anti rabbit IgG (H+L) | WB | 1:10000 | ZSGB-Bio | ZB-2301 |
| HRP-labeled goat anti mouse IgG (H+L) | WB | 1:10000 | ZSGB-Bio | ZB-2305 |
| Biotin-labeled goat anti rabbit IgG (H+L) | WB | 1:2000 | Yeasen | 33103ES60 |
| HRP-labeled Streptavidin | WB | 1:5000 | Beyotime | A0303 |

Table S2. Insertion sequences in pGL4.13-Nfkbiz-promoter and pGL4.13-Nfkbiz-3UTR

| Plasmid: pGL4.13-Nfkbiz-promoter | Insertion sequence: the promoter of mouse *Nfkbiz* |
| --- | --- |
| 5’-ATTTTCCTGCAAACGCTTTAGGGCCATCCTTTAGGGCTCATCTCATTCTTTGCGAAGACTTCCCCCTTCTCCCCCTCTCCACAAGAAAGTCTCTTTTTAGGCTGTTCACTGAACTTTGTACCTCTCACGATGTTTAATCATAGTCAGTTTCTTTTCATCGGTGTGTGTATACCTTATCCCCACGTGTTAAGATGTTACTTGAAAATTTAGACTTAATCATTCATTCTTTCTCTCCCTTAAAAATAAACTTCCCTTGACGGTTCGCTAGTTGGATTTGCGGCGGAAGTATAAACATTGAAAAAAGTATTCAAGGTAGAGGCATCAAAATCCCTGTGTTTATCAGGACTAGGTCAAGTTGTGCAGAATAAAACGTTACATAATACGTAAAAAATAATATAAAACATACAAAGAATATACACGCATATATGCGTATTCATCATTTTGCTAGAAGCTTTTTTCTAGTATTGAAGGATACGAAAGGGTAGTCATTGCTGTTTATTCTAATACTGTACTAAAAAAAAAAAAAAGATTGCTTATGGCCCAGATATGAAGTTAGAGAAACTGCAAGAGCTCTGAAATTGAGTCCAGGCCAGAAAGTCCGCGGGCAACCGCTTCCAGGTTTGGAGGCGGGCTAAATGTTGGAGGCAAGGGAGGAGACTGAACTCTGCACGCACCGCTCTGGCTTCCCACTCCCCGAACTGGGCGGACCTGTTCTTGCAGCAGCCCGGGGCTCCCGCTCGCCCCCGGGCATCGCAGCCCCTGCGCAGACAGCGGTTGCCGCGGACCCCGGGCCCCTGGGGATGCGGTGCGTCCAGCGAAGGCCAACCGGGGAAACTTGGAGCTGGCCCCGACGGGGGTTCGGGGCGCGGGTGCTAGCCGGGGTTTCCAGGTGAGCCGCCGGAAAACAACCGGTCGGCCCGGGCGGGGCGGGGCGAGGGCGGAGGGCACATTGCCTCATCCCGGGCATTTTACTGGAAACTAGACACATCCGGAGGCGGGGCGGGAGGCAGCCCAGGCGCCGCCTCACCGGGCGGAGCGCGGAGGAGGAGGTCAGTTCATTTAAATAGCCCGGTCCCCTCCGGGCGCGCACGTACTAGTCGCTCTGCCGCAGCCAACCGCGTAGCCATCCGGTGGCGCAGGTGTCTGGGGACCCCGAGCGCCTAGCCTGGGAGC-3’ | |
| Plasmid: pGL4.13-Nfkbiz-3UTR | Insertion sequence: the 3’-UTR elements of mouse *Nfkbiz* |
| 5’-GTCCACTGGCGTGGAGCCTGGTTCAGCGACACTCACTGTCAGTTAGGCAGTCCTGATGTATCTGTACATAGACCATTTGCCCTGTATTGGCAAATGTAAGTTGTTTCTATGAAACAAACCCATTTAGTTCACTATTATATAGTGGATTATATTAAAAGAAAAGAAGACAGATATCTAATTTTCTTGGCAGATTTGCGTATTTCATACCCAGGTATCTGGGATCTATATATCTGAATTTGATCTTGAATGGTAAAATTACCTTCGATAACCAGTAGCTTT-3’ | |

**Table S3 Quantitative PCR primers used in this study**

| Gene | Primer sequence |
| --- | --- |
| Mouse *Tnf* | Forward 5’-CACGTCGTAGCAAACCACC-3’  Reverse 5’-GGTGAGGAGCACGTAGTCG-3’ |
| Mouse *Il1b* | Forward 5’-TGCCACCTTTTGACAGTGATG-3’  Reverse 5’-TGATGTGCTGCTGCGAGATT-3’ |
| Mouse *Il6* | Forward 5’-CCTCTCTGCAAGAGACTTCCAT-3’  Reverse 5’-ACAGGTCTGTTGGGAGTGGT-3’ |
| Mouse *Nos2* | Forward 5’-GGTGAAGGGACTGAGCTGTT-3’  Reverse 5’-ACGTTCTCCGTTCTCTTGCAG-3’ |
| Mouse *Fosb* | Forward 5’-GATCGACTTCAGGCGGAAAC-3’  Reverse 5’-TGGCAAATCTCTCACCTCGG-3’ |
| Mouse *Nfkbid* | Forward 5’-CTTCCTTCTGTCTTCCCACACAC-3’  Reverse 5’-AATGAGTATGGCCTGGCTCTGC-3’ |
| Mouse *Cebpd* | Forward 5’-AGAACCCGCGGCCTTCTAC-3’  Reverse 5’-ATGTAGGCGCTGAAGTCGAT-3’ |
| Mouse *Nfkbiz* | Forward 5’-GAAGTCCCGAGGCCAACC-3’  Reverse 5’-AAGTAAGCCAGGTTGAGCGG-3’ |
| Mouse *Atf3* | Forward 5’-GTGCCTGCAGAAAGAGTCAGA-3’  Reverse 5’-CTTCCGGTGTCCGTCCATTC-3 |
| Mouse *Junb* | Forward 5’-CTTTGCGGACGGTTTTGTCA-3’  Reverse 5’-GCTGAGGTTGGTGTAGACGG-3’ |
| Mouse *Irf1* | Forward 5’-CAGCATCTCGGGCATCTTTC-3’  Reverse 5’-GTGATTGGCATGGTGGCTTTG-3’ |
| Mouse *Hmox1* | Forward 5’-CAGAAGAGGCTAAGACCGCC-3’  Reverse 5’-GCAGTATCTTGCACCAGGCTA-3’ |
| Mouse *Nqo1* | Forward 5’-CCATGTACGACAACGGTCCT-3’  Reverse 5’-GCAGGATGCCACTCTGAATC-3’ |
| Mouse *Actb* | Forward 5’-CGCAGCCACTGTCGAGTC-3’  Reverse 5’-GTCATCCATGGCGAACTGGT-3’ |
